# Mesenchymal Stromal Cell-Derived Microvesicles Regulate an Internal Pro-Inflammatory Program in Activated Macrophages

**DOI:** 10.3389/fimmu.2017.00881

**Published:** 2017-07-31

**Authors:** Juan S. Henao Agudelo, Tarcio T. Braga, Mariane T. Amano, Marcos A. Cenedeze, Regiane A. Cavinato, Amandda R. Peixoto-Santos, Marcelo N. Muscará, Simone A. Teixeira, Mario C. Cruz, Angela Castoldi, Rita Sinigaglia-Coimbra, Alvaro Pacheco-Silva, Danilo C. de Almeida, Niels Olsen Saraiva Camara

**Affiliations:** ^1^Department of Medicine, Division of Nephrology, Federal University of São Paulo, Sao Paulo, Brazil; ^2^Department of Immunology, Institute of Biomedical Sciences, University of São Paulo, Sao Paulo, Brazil; ^3^Department of Pharmacology, Institute of Biomedical Sciences, University of São Paulo, Sao Paulo, Brazil; ^4^Electron Microscopy Center, Federal University of São Paulo, Sao Paulo, Brazil; ^5^IEP, Albert Einstein Hospital, Sao Paulo, Brazil; ^6^Laboratory of Renal Pathophysiology, Department of Medicine, School of Medicine, University of São Paulo, Sao Paulo, Brazil

**Keywords:** mesenchymal stromal cells, microvesicles, macrophages, immunomodulation, acute peritonitis

## Abstract

Mesenchymal stromal cells (MSCs) are multipotent cells with abilities to exert immunosuppressive response promoting tissue repair. Studies have shown that MSCs can secrete extracellular vesicles (MVs-MSCs) with similar regulatory functions to the parental cells. Furthermore, strong evidence suggesting that MVs-MSCs can modulate several immune cells (i.e., Th1, Th17, and Foxp3^+^ T cells). However, their precise effect on macrophages (Mϕs) remains unexplored. We investigated the immunoregulatory effect of MVs-MSCs on activated M1-Mϕs *in vitro* and *in vivo* using differentiated bone marrow Mϕs and an acute experimental model of thioglycollate-induced peritonitis, respectively. We observed that MVs-MSCs shared surface molecules with MSCs (CD44, CD105, CD90, CD73) and expressed classical microvesicle markers (Annexin V and CD9). The *in vitro* treatment with MVs-MSCs exerted a regulatory-like phenotype in M1-Mϕs, which showed higher CD206 level and reduced CCR7 expression. This was associated with decreased levels of inflammatory molecules (IL-1β, IL-6, nitric oxide) and increased immunoregulatory markers (IL-10 and Arginase) in M1-Mϕs. In addition, we detected that MVs-MSCs promoted the downregulation of inflammatory miRNAs (miR-155 and miR-21), as well as, upregulated its predicted target gene SOCS3 in activated M1-Mϕs. *In vivo* MVs-MSCs treatment reduced the Mϕs infiltrate in the peritoneal cavity inducing a M2-like regulatory phenotype in peritoneal Mϕs (higher arginase activity and reduced expression of CD86, iNOS, IFN-γ, IL-1β, TNF-α, IL-1α, and IL-6 molecules). This *in vivo* immunomodulatory effect of MVs-MSCs on M1-Mϕs was partially associated with the upregulation of CX3CR1 in F4/80^+^/Ly6C^+^/CCR2^+^ Mϕs subsets. In summary, our findings indicate that MVs-MSCs can modulate an internal program in activated Mϕs establishing an alternative regulatory-like phenotype.

## Introduction

Mesenchymal stromal cells (MSCs) are multipotent cells with mesodermal differentiation capacity into adipocytes, osteoblasts, and chondroblasts (1). MSCs were originally described in bone marrow, but currently these cells can be isolated from various vascularized tissues including the adipose tissue (2). Therapeutically, MSCs are able to migrate toward injured tissues exerting anti-inflammatory and anti-apoptotic functions that consequently promote tissue repair (3). In addition, MSCs also have low immunogenicity when transplanted into patients, indicating their use as promising candidate for cell-based therapies (4). With regard to its regenerative properties, there are more than 600 protocols using MSCs in several diseases which include mainly orthopedic, cardiovascular, and autoimmune disorders (see in https://www.clinicaltrials.gov).

The MSCs ability to support tissue repair has been primarily associated with its capacity to modulate the immune response promoting a pro-resolution microenvironment (5). Extensive evidence has shown that MSCs can modulate dendritic cells, T lymphocytes (CD4 and CD8), natural killer, B cells, and more recently macrophages (Mϕs) (6–9). In light of this evidence, it has been reported that MSCs are effective to reprogram and educate classically activated macrophages (M1-Mϕs) to an alternative regulatory phenotype (M2-Mϕs) (10). The main mechanism behind this modulation of Mϕs by MSCs involves mainly the release of soluble immunomodulatory agents such as TSG-6, PGE2, and the upregulation of IDO enzymatic activity (11). Furthermore, it was observed that Mϕs in contact with MSCs exhibit downregulation of pro-inflammatory markers (i.e., TNF−α, IL-1α, IL-6, IL-1β, IL-12p70, MCP-1, iNOS, and CD86) and upregulation of anti-inflammatory molecules such as IL-10, arginase-1, and CD206 (10–12).

Additionally, other studies have shown that MSCs can secrete extracellular vesicles (EVs) with similar immunoregulatory and regenerative functions found in parental cells (13, 14). EVs can be defined as a heterogeneous population of vesicles derived from cell membranes that can be classified into: apoptotic bodies (1–5 μm), microvesicles (MVs) (100–1,000 nm), and exosomes (EXOs) (40–100 nm) according to their size, biogenesis, and density (15, 16). Interestingly, pre-clinical therapies based on EXOs or MVs derived from MSCs have been effective in models of cardiovascular, neurologic, and kidney diseases (13, 17, 18). Furthermore, in the context of inflammation, there is strong evidence that MSCs-derived EVs (EVs-MSCs) can inhibit Th17 cells, induce the conversion of Th1 to Th2 response and promote the proliferation of regulatory Foxp3^+^ T cells (14, 19). Altogether, these findings suggest that EVs-MSCs possess a potent immunoregulatory capacity on lymphocytes and probably in other immune cell subsets involved in the inflammatory response. However, the precise effect of MSCs-derived microvesicles (MVs-MSCs) on the immunobiology of Mϕs remains unexplored. Thus, considering the important role of Mϕs in tissue regeneration, the aim of this study was to evaluate the immunoregulatory effect of MVs-MSCs on classically activated M1-Mϕs.

## Materials and Methods

### Animals

Around 8- to 10-week-old C57BL/6 male mice (20–25 g) were obtained from the Center for Experimental Models Development for Medicine and Biology (CEDEME) of the Federal University of São Paulo (UNIFESP). Additionally, B6:129P CX3CR1^+/GFP^ and B6:129 (Cg) CCR2^+/RFP^ mice were obtained from Jackson Laboratory (Bar Harbor, ME, USA), and these inbred strains were backcrossed to generate CCR2^+/RFP^ CX3CR1^+/GFP^ mice. All mice were housed in a clean room with constant temperature (22°C) and a light/dark cycle of 12/12 h. The mice received a solid diet and filtered water *ad libitum*. All animal protocols and experiments were approved by the Ethics Committee on Animal Experimentation of UNIFESP with accession number CEP 685598.

### Isolation and Characterization of Adipose-Derived MSCs

Adipose-derived MSCs were isolated from epididymal fat of C57BL/6 mice (*n* = 10) as previously published (20). Briefly, adipose tissue was washed with PBS-containing penicillin/streptomycin 50 U/mL (Gibco, Thermo, USA) and minced with surgical scissors. Subsequently, the tissue was incubated with collagenase type IA solution 0.075% (Sigma-Aldrich, USA) at 37°C for 30 min. After digestion, the enzyme activity was neutralized by adding DMEM Low Glucose culture medium (Gibco) with 10% fetal bovine serum (FBS, Hyclone, Thermo Fisher Scientific). The digested material was centrifuged at 300 *g* for 10 min, and the pellet was subjected to red blood cells lyse solution treatment with NH_4_Cl 0.83%. Then, the cell pellet was twice washed with PBS and centrifuged at 300 *g* for 5 min. The cells were incubated at 37°C in humid atmosphere with 5% CO_2_ with DMEM Low Glucose with antibiotics (penicillin/streptomycin 100 U/mL) and 10% FBS. At confluence of 80–90% of the cell monolayer, MSCs were trypsinized (0.05%, Trypsin-EDTA, Gibco) and further expanded to 10–12 passages. The immunophenotypic characterization and of multilineage differentiation of MSCs was performed according to previous works from our group (20, 21).

### Isolation of MVs-MSCs

Mesenchymal stromal cells were expanded until 80% of confluence with DMEM low glucose (10% FBS). At high density confluence, culture growth medium was changed to serum-free DMEM low glucose. After 48 h, the conditioned medium was collected and centrifuged at 2,000 *g* for 30 min at 4°C to remove cells and cellular debris. Then, the conditioned medium was subjected to ultracentrifugation at 100,000 *g* for 2 h at 4°C, using a SW28 rotor (Optima L-90K, Beckman Coulter, USA). Finally, the pellet containing MVs-MSCs was re-suspended in PBS and stored at −80°C. MVs-MSCs protein content was quantified by Pierce method (Thermo Fisher Scientific) and characterized using flow cytometry (BD LSRFortessa, BD biosciences) and nanoparticle tracking analysis (NTA)-based methods (NanoSight, Malvern, UK). Transmission electron microscopy was performed using protocols established in our center of facilities for electron microscopy at Federal University of São Paulo (CEME).

### MVs-MSCs NTA

Microvesicles-MSCs were analyzed by NTA using NanoSight LM10 (NanoSight, UK). The laser was set at 405 nm and the speed of the high-sensitivity camera shutter was set at 30.01 ms (OrcaFlash2.8). The videos were analyzed using NTA software (version 2.3 NanoSight), and recordings were made for 30 s per sample. All samples were diluted in ultrapure PBS (Gibco, Thermo, USA) to achieve a concentration of 2 × 10^8^ and 9 × 10^10^ particles/mL. Samples were administered and registered under controlled flow, using NanoSight syringe pump system. Finally, histograms were generated as results of NTA analyzes to evaluate particle concentration and average size of MVs-MSCs.

### MVs-MSCs Electron Microscope Analysis

After ultracentrifugation, MVs-MSCs containing pellet was resuspended in 100 μL of 2% buffered formaldehyde solution. Then, drops of 20 μL were placed on parafilm in a flat surface. Formvar-carbon-coated electron microscopy grids were placed on the drops for 10 min for MVs adsorption. Grids were washed with 0.1M sodium cacodylate buffer, post fixed in 1% buffered glutaraldehyde, and washed with deionized water. Negative-positive staining was performed according to Théry et al. (22). Briefly, grids were contrasted in oxalic uranyl, then embedded in a mixture of saturated uranyl and methylcellulose 2% (1:9) on ice. Fluid excess was gently blotted on filter paper and air dried. Images acquisition and observation was run under a JEOL 1200 EX II (Japan) transmission electron microscope at 80 kV.

### Isolation and Polarization of Bone Marrow-Derived Macrophages (BMDM)

Femurs and tibia of C57BL/6 male mice were dissected, and the epiphyses were surgically removed with scissors to perform internal flushing with sterile PBS. The cells thus obtained from bone marrow were filtered using Cell Steiner 70 μm filter (Corning, USA), and the flow-through cells were twice centrifuged and washed at 300 g for 5 min. Subsequently, cells were lysed with 0.83% NH_4_Cl (3 min/4°C) and cultured in DMEM high glucose medium (Gibco) containing 10% FBS (Gibco) and 30% of supernatant medium from L929 cells (ATCC, Cell Lines) (23). The culture medium was refreshed on day 4 and subsequently maintained until day 7 to promote BMDM differentiation. On day 7, 2 × 10^5^ Mϕs/cm^2^ were polarized to M1 by 5 ng/mL IFN-γ (R&D Systems, USA) and 50 ng LPS/mL (E. coli-LPS, Sigma-Aldrich, USA) incubation for 24 h. In contrast, M2 polarization was induced by 10 ng IL-4/mL and 10 ng IL-13/mL (R&D Systems) for 24 h.

### M1-Mϕs Coculture Assays with MSCs or MVs-MSCs

M1-Mϕs were seeded at a concentration of 2 × 10^5^ Mϕs/cm^2^ and co-cultivated with 1 × 10^5^ MSCs in cell-to-cell contact systems (1:2) for 48 h in DMEM high glucose with 10% FBS and samples collected with 72 h of total incubation (Figure S1E in Supplementary Material). On the other hand, in experiments using MVs-MSCs, we treated M1-Mϕs (2 × 10^5^ Mϕs/cm^2^) with serial doses of 4.19 × 10^9^ particles of MVs-MSCs every 8 h for a total period of 48 h in DMEM high glucose with 10% FBS and samples collected with 72 h total incubation (Figure S1F in Supplementary Material). As control, M1-Mϕ was treated with vehicle PBS (Gibco, Thermo, USA) using similar conditions as the above mentioned. M1-Mϕs control was cultured for 48 h in DMEM high glucose with 10% SFB but without stimulus or MSCs/MVs-MSCs. Finally, unstimulated Mϕs were maintained in culture for 72 h as an internal control of M1 polarization. All experiments conducted with MSCs or MVs-MSCs in coculture assays with Mϕs were evaluated after 72 h of incubation (Figures S1E,F in Supplementary Material). In addition, to observe MVs-MSCs internalization into M1-Mϕs, a single dose of MVs-MSCs (4.19 × 10^9^ particles) were pre-labeled with PKH26 (Sigma-Aldrich) and administered onto monolayers of 2 × 10^5^ Mϕs/cm^2^. Images and videos were acquired during 4 h in LSM 880 Laser Scanning Microscope Zeiss (Carl Zeiss, Germany).

### Flow Cytometry Analyses

For Mϕs multi-step analyses by flow cytometry (FACS), the following markers were used: F4/80, CD11b, CD86, CCR7, CD206, Ly6C (BioLegend, USA) Siglec-F (BD biosciences, USA), and iNOS (BD biosciences, USA). Before staining, Mϕs were incubated with mouse Fc Block (BD Biosciences, USA) for 5 min at 4°C to prevent non-specific antibody interactions. Cells were then washed with staining buffer (BD Biosciences, USA) and centrifuged at 300 *g* for 5 min. For surface markers detection, Mϕs were incubated with staining buffer containing the respective mix of antibodies for 30 min at 4°C, protected from light. Next, cells were washed and acquired by FACSCanto II our LSRFortessa (BD biosciences, USA). For intracellular staining, the cells were previously incubated with GolgiStop (BD biosciences, USA) by 4 h following the manufacturer’s recommendations. Subsequently, for iNOS detection, Mϕs were treated with fixation/permeabilization solution (BD Biosciences, USA) and incubated with the respective antibody. Next, cells were washed and samples were acquired by FACS. Compensation controls were performed by CompBeads (BD Biosciences, USA), and acquisition controls were based on the fluorescence minus one control method. For MVs-MSCs immunophenotyping, the following antibodies were used: CD90, CD105, CD73, CD31, CD45, CD44 (BD biosciences, USA), and primary CD9 antibody (Abcam, USA), which was subsequently conjugated with Alexa fluor 488 (Abcam, USA). To detect surface antigens in MVs-MSCs, the particles were incubated with the respective antibody in single color staining for 30 min at 4°C. MVs-MSCs were then ultracentrifuged and washed twice in PBS, and subsequently analyzed by FACS. MVs-MSCs populations were set up with the assistance of sizes defined beads (3.8 and 7 μm) and additionally with annexin V antibody (BD biosciences, USA). All data were collected by BD FACSDiva and analyzed using FlowJo V10 software (Tree Star, USA).

### *In Vivo* Model of Peritonitis Induced by Thioglycollate

Thioglycollate medium 3% (Sigma-Aldrich) was prepared in sterile conditions and protected from light at least 6 months before experiments. Mice were injected intraperitoneally (IP) with 2 mL of thioglycollate solution and euthanized on day 4. Mice treated with MVs-MSCs were injected IP with four doses of MVs-MSCs (8.38 × 10^9^ particles) at 0, 24, 48, and 72 h. In contrast, control mice were treated at the same time-points but using vehicle (PBS). Finally, on the 4th day after thioglycollate infusion, an peritoneal lavage was performed with 5 mL of cold sterile PBS. The peritoneal exudate was centrifuged at 300 *g* for 5 min and cells were processed by FACS and the supernatant was analyzed by MILLIPLEX (Merck Millipore, USA). During the standardization of the model of peritonitis, washes at 24, 48, 72, and 96 h were performed to identify the cell cluster with GR1, CD19, and CD11b phenotype by FACS (Figures S4A,B in Supplementary Material). Furthermore, the predominant presence of Mϕs in the last day of the induction of peritonitis was confirmed by morphological analysis (HE) and immunohistochemistry for CD11b (Figure S4C in Supplementary Material).

### Cytokine Assessment in Intraperitoneal Lavage

The concentrations of IFN-γ, IL-1α, IL-1β, TNF-α, IL-6, and IL-10 cytokines in the mouse peritoneal lavage were measured using MILLIPLEX MCYTOMAG-70K kit (Millipore Corporation, USA). Controls and samples were processed according to manufacturer’s instructions. The sample detection was performed by Luminex SD device (Luminex, USA) using the software MILLIPLEX Analyst (Millipore Corporation).

### Gene and miRNAs Expression

Total RNA derived from Mϕs was isolated using Trizol Reagent (Thermo Fisher Scientific), and its concentration and purity were determined using Nanodrop (Thermo Fisher Scientific). The cDNA was then synthesized using M-MLV reverse transcriptase (Promega, USA) following the manufacturer’s specifications. Subsequently, qPCR reactions were performed using the 7300 Real-Time PCR System (Applied Biosystems, USA) with TaqMan or SYBR green assays. For the TaqMan method, the following probes were used: IL-10 (Mm00439616_m1), IL-6 (Mm00446190_m1), Nos2 (Mm01309902_m1), IL-1β (Mm00434228_m1), irf4(Mm01165980_m1), Arg1 (Mm00475990_m1), Hprt (Mm00446968_m1). For the SYBR green assays, these previously validated pair primers were used: Fizz1 5′-TCCCAGTGAATACTGATGAGA-3′ and 5′-CCACTCTGGATCTCCCAAGA-3′; Ym1 5′-GGGCATACCTTTATCCTGAG-3′ and 5′-CCACTGAAGTCATCCATGT-3′; SOCS3 5′-GGGTGGCAAAGAAAAGGAG-3′ and 5′-GTTGAGCGTCAAGACCCAGT-3′; SOCS1 5′-ACCTTCTTGGTGCGCGAC-3′ and 5′-AAGCCATCTTCACGCTGAGC-3′; CXCL9 5′-TGCACGATGCTCCTGCA-3′ and 5′-AGGTCTTTGAGGGATTTGTAGTGG-3′. The results were analyzed by SDS Software (Applied Biosystems, USA), and gene expression was normalized by the expression of the internal control gene Hprt. The analysis of each sample was performed in triplicates and data were expressed by relative quantification method URE (10,000/2^ΔCt^) (24). Furthermore, for miRNAs analysis, the total RNA was extracted from Mϕs using the miRNeasy Mini Kit (Qiagen, DEU) according to manufacturer’s instructions. Around 2 μg of RNA was used to amplify mature miRNAs present in the total RNA. This technique was performed using the miScript II RT kit (Qiagen) according to manufacturer’s instructions. The miR-155, miR-21, and miR-146a (Primer Assays, Qiagen) primers were used for miRNAs expression. The qPCR reaction for miRNAs was performed with SYBR Green PCR kit (Qiagen) using 7300 Real-Time PCR System.

### Enzymatic Activity of Arginase

The enzymatic activity of arginase was measured using an *in house* method. Total protein lysate from Mϕs was diluted 1:10 in 50 mM Tris-HCl buffer pH 7.4 containing 0.5 M PMSF and protease inhibitors. Then, 120 μL of each sample was transferred to 1.5 mL tubes and 120 μL of MnCl_2_ 10 mM were added to each tube, which were further heated to 55°C for 10 min. After incubation, the tubes were placed on ice and 240 μL of L-Arg (0.5 M) were added. Then each sample was divided into additional four 0.6 mL tubes with 100 μL per tube. Two tubes were kept at 37°C for 1 h and the others left in ice for 1 h (control reaction). After this second incubation period (1 h), all tubes were taken to a fumes hood and 500 μL of the mix of acid solution (H_2_SO_4_:H_3_PO_4_:H_2_O) (1:3:7) were added, as well as 25 μL of ISPF 9%, always protected from light. Subsequently, tubes were sealed with parafilm. Samples were heated for 45 min at 100°C in a water bath, with 200 μL of each reaction product then pipetted on an ELISA plate. Samples were read in a spectrophotometer at 540 nm. The final results were expressed in nmol of protein (urea/min/mg).

### Dosage of Nitric Oxide (NO)

Nitric oxide estimation was performed by indirect colorimetric Griess method. First, a standard curve was constructed with NaNO_2_ using concentrations of 5, 10, 30, and 60 μM (Sigma-Aldrich, USA). Then, 50 μL of Mϕs cell lysate was incubated with 50 μL of Griess reagent for 1 h. After incubation, the plates were analyzed spectrophotometrically at 550 nm. The results were expressed in micromolar and normalized by the total protein concentration (μM/μg).

### Statistical Analysis

For statistical analyses to compare two samples we used Student’s *t*-test, and in analyses that comprised more than two independent samples one-way ANOVA was performed followed by *post hoc* Tukey test. The results are presented as mean and SD for parametric variables. Differences were considered significant if *P* < 0.05. Statistical analyses and graphs were performed with GraphPad Prism version 5.0 (GraphPad Software, USA).

## Results

### Isolation and Characterization of MVs-MSCs

Previous studies have clearly demonstrated the effectiveness of MSCs to educate and reprogram M1-Mϕs and M2-Mϕs (11, 25). Considering this perspective, we tested the ability of adipose-derived MSCs to regulate M1-Mϕs (Figure S1 in Supplementary Material). Using the gate exclusion strategy with Mϕs specific marker (F4/80), it was possible to discriminate the Mϕs and MSCs independent sub-populations (Figure S1A in Supplementary Material). Further coculture analysis confirmed that MSCs decreased the expression of costimulatory molecules (CD86/B7-2) and chemokine receptor (CCR7) in M1-Mϕs previously stimulated with LPS/IFN-γ (Figures S1B,C in Supplementary Material). Additionally, the MSCs also increased the mannose receptor (CD206) frequency, a molecule associated with M2 profile, in M1-Mϕs cocultures (Figure S1D). Interestingly, unstimulated Mϕs presented similar behavior to MSCs-modulated M1-Mϕs (Figures S1B,D). These findings indicate that MSCs in direct contact with M1-Mϕs can promote a prominent immunomodulatory effect. According to these results, we isolated MVs from adipose-derived MSCs supernatants to investigate if MVs-MSCs could assist a similar regulatory function as that observed for its parental cells.

**Figure 1:**
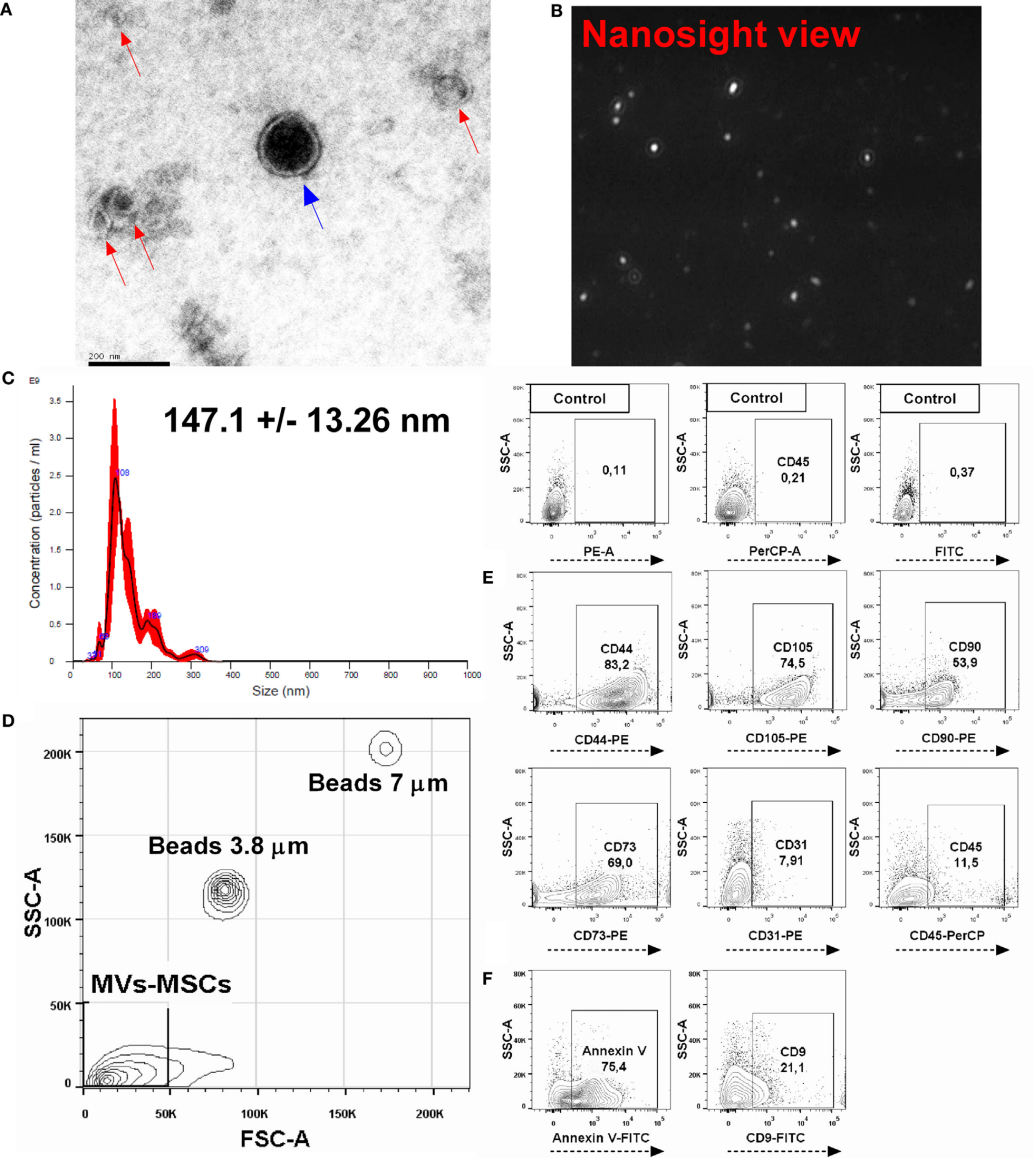
Characterization of MSCs-derived microvesicles (MVs-MSCs). **(A)** Electron micrographs of MVs-MSCs showing a circular and double-membrane structure; in the center of the field an MV (blue arrow) is observed, and other vesicles distributed around it compatible with exosomes (red arrows). **(B)** MVs-MSCs viewed by nanosight; **(C)** analysis of size and concentration of MVs-MSCs with nanoparticle tracking analysis; **(D)** FACS parameter analysis (SSC, complexity and FSC, size) of MVs-MSCs populations using defined beads; **(E)** investigation of MSCs immunophenotype panel in MVs-MSCs suspension, and **(F)** expression of annexin V and CD9 in MVs-MSCs surfaces. In general, MSCs-derived particles presented features of the parental cells with classical MVs profile (*P < 0.05, **P < 0.01, ***P < 0.001).

Initially, we characterized the MVs-MSCs population and defined its size, morphology, and immunophenotypic characteristics. Transmission electron microscopy analysis revealed that MVs-MSCs exhibit double membrane structures and present spheroid shape with a diameter inferior to 200 nm (Figure 1A). Additionally, to confirm size and concentration of the MVs-MSCs, we performed a NTA using the NanoSight equipment. This assay showed that purified MVs-MSCs samples had a size around 147.1 ± 13.26 nm and an approximate concentration of 1.0 × 10^11^ particles/mL (Figures 1B,C). Finally, we investigated if markers associated with MSCs also could be found in MVs-MSCs. We used predetermined particle size (beads with 3.8 and 7 μm) to discriminate the MVs-MSCs population location in FACS workflow (Figure 1D). MVs-MSCs constitutively expressed the classical MSCs antigens such as CD44, CD105, CD90, CD73, but not endothelial (CD31) and hematopoietic (CD45) molecules (Figure 1E). Additionally, as an internal control, we found that MVs derived from macrophages (MVs-Mϕs) do not express classical MSCs antigens (i.e., CD73, CD90, and CD105) on its surface, suggesting the high specificity of our MVs-MSCs immunophenotypic profile (Figure S2 in Supplementary Material). Moreover, MVs-MSCs presented annexin V (microvesicle marker) and tetraspanin (CD9, an EXOs marker) surface expression (Figure 1F). In summary, our structural and immunophenotypic analysis suggest that our EVs-MSCs population, defined here as MVs-MSCs, can be comprised as a heterogeneous subpopulation predominantly enriched with MVs (particles with 100–1,000 nm).

### MVs-MSCs Interacts with M1-Mϕs to Regulate Activation, Oxidative Stress, and Secretory Profile

**Figure 2:**
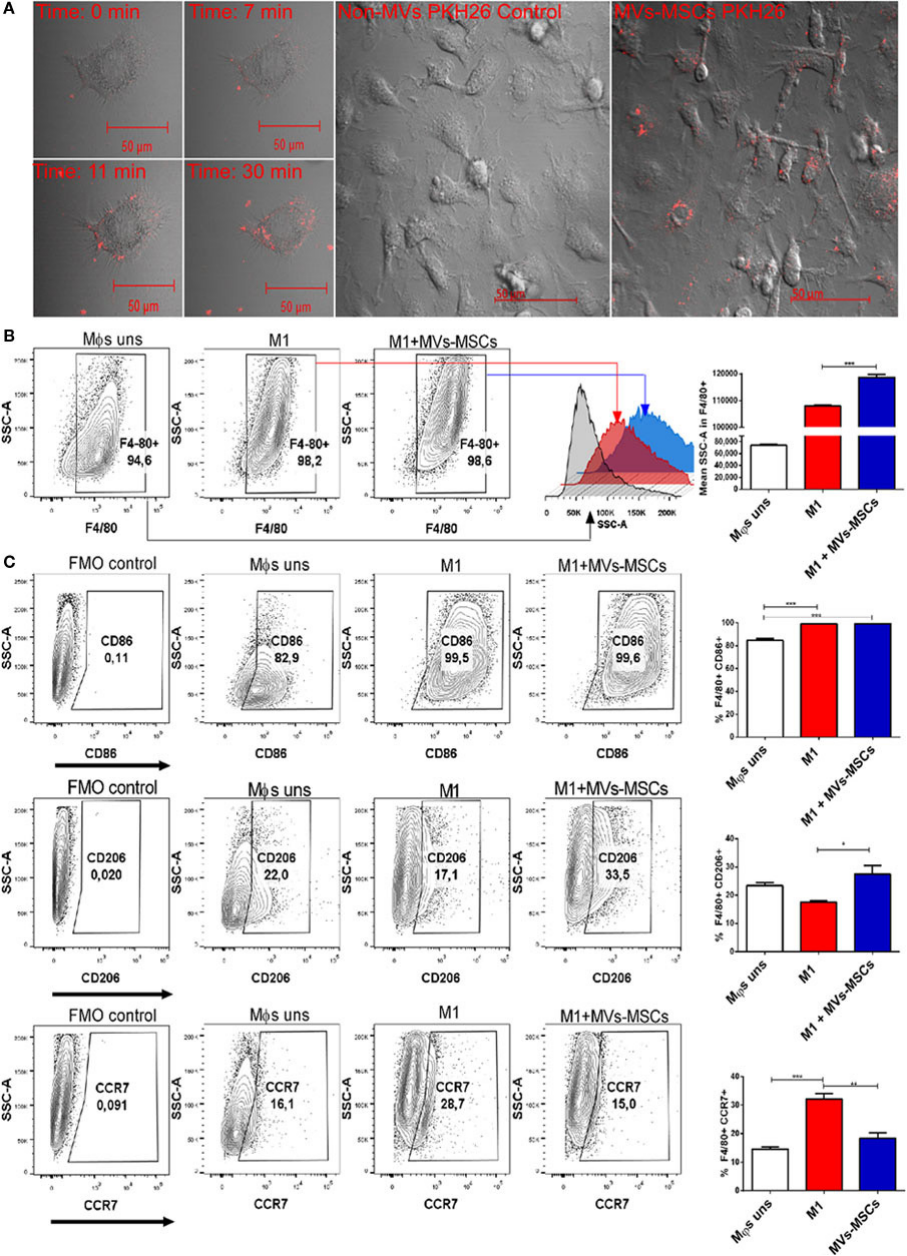
Incorporation of MVs-MSCs in M1-Mϕs and expression of pro-inflammatory and anti-inflammatory surfaces markers. **(A)** PKH26 red labeled MVs-MSCs incorporation into M1-Mϕs; **(B)** macrophage internal granularity; and **(C)** expression of pro-inflammatory and anti-inflammatory surface molecules in M1-Mϕs and M0-Mϕs treated or not with MVs-MSCs (*P < 0.05, **P < 0.01, ***P < 0.001).

To investigate the capacity of MVs-MSCs to interact and regulate the pro-inflammatory profile of M1-Mϕs, we initially isolated bone marrow-derived Mϕs and extensively differentiated and characterized these cells to M1 and M2 phenotypes (Figure S3 in Supplementary Material). We observed by fluorescent confocal microscopy the ability of MVs-MSCs to associate/incorporate into M1-Mϕs. Live cell imaging showed that MVs-MSCs pre-labeled with PKH26 (red fluorescent dye) had the ability to attach and partially incorporate into the M1-Mϕs cytoplasm right after their administration to M1-Mϕs (i.e., in less than 30 min) (Figure 2A). Visible MVs-MSCs aggregates were detected on M1-Mϕs surface or in intracytoplasmic space during the timecourse (Figure 2A) (Video S1 in Supplementary Material). Moreover, we also confirmed that a single dose of MVs-MSCs suspension can be rapidly incorporated into a confluent macrophage culture (Figure 2A) (Video S3 in Supplementary Material). As a control, the PKH26 red dye alone did not incorporate into M1-Mϕs (Figure 2A) (Video S2 in Supplementary Material).

To confirm the transfer of vesicles into the cytoplasm, we evaluated granularity in M1-Mϕs treated with serial doses of MVs-MSCs. Pre-treated M1-Mϕs had their side scatter (SSC, granularity) parameter increased when compared with untreated and activated M1-Mϕs (Figure 2B). This suggests that M1-Mϕs may incorporate MVs-MSCs and subsequently enhance its internal granularity, probably by the transfer or indirect production of bioactive molecules such as mRNA, miRNAs, proteins, and lipids (26).

**Figure 3:**
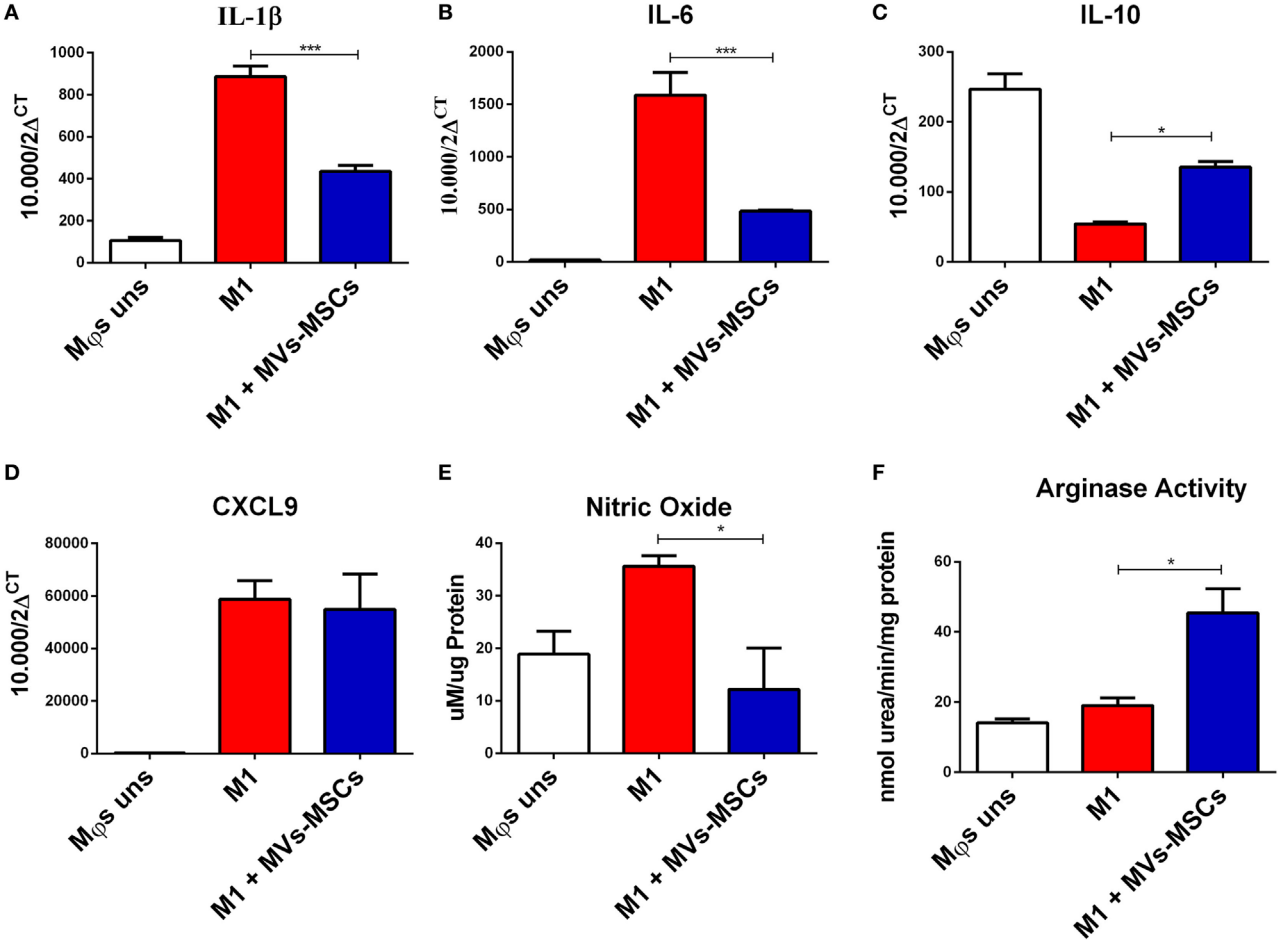
Global expression of inflammation and oxidation-associated molecules in M1-Mϕs treated or not with MVs-MSCs. **(A)** IL-1β expression; **(B)** IL-6 expression; **(C)** IL-10 expression; **(D)** CXCL9 expression; **(E)** nitric oxide production; and **(F)** activity of arginase in M1-Mϕs. MVs-MSCs are shown to efficiently modulate the pro-inflammatory and oxidative status in M1-Mϕs (*P < 0.05, **P < 0.01, ***P < 0.001).

We then investigated the ability of MVs-MSCs to modulate pro-inflammatory markers in M1-Mϕs. The treatment with MVs-MSCs decreased M1-related molecules such as CCR7, but not CD86, a classical marker of activation (Figure 2C). On the other hand, MVs-MSCs administration increased the expression of M2-associated markers in M1-Mϕs such as mannose receptor (CD206) (Figure 2C). Furthermore, MVs-MSCs potentially reduced the mRNA expression of inflammatory cytokines such IL-1β and IL-6 in M1-Mϕs, while increasing anti-inflammatory molecules such as IL-10 (Figures 3A–C). Interestingly, the expression of CXCL9, a strong chemoattractant protein, remained unaltered in M1-Mϕs treated or not with MVs-MSCs (Figure 3D). Finally, we attempted to show the MVs-MSCs ability to reduce oxidative stress in M1-Mϕs. We identified that MVs-MSCs treatment increased arginase activity (M2 marker) in M1-Mϕs and reduced endogenous NO production (M1 marker) (Figures 3E,F). In most experiments, unstimulated Mϕs presented a pattern similar to the observed in MVs-MSC-treated M1-Mϕs. Thus, our results suggest that MVs-MSC were effective to reduce activation, oxidative stress, chemokine receptor expression, and pro-inflammatory cytokines on M1-Mϕs, thereby inducing a switch from M1 to a M2-like phenotype.

### MVs-MSCs Exerts a Down-Regulation of Pro-inflammatory miRNAs in M1-Mϕs

**Figure 4:**
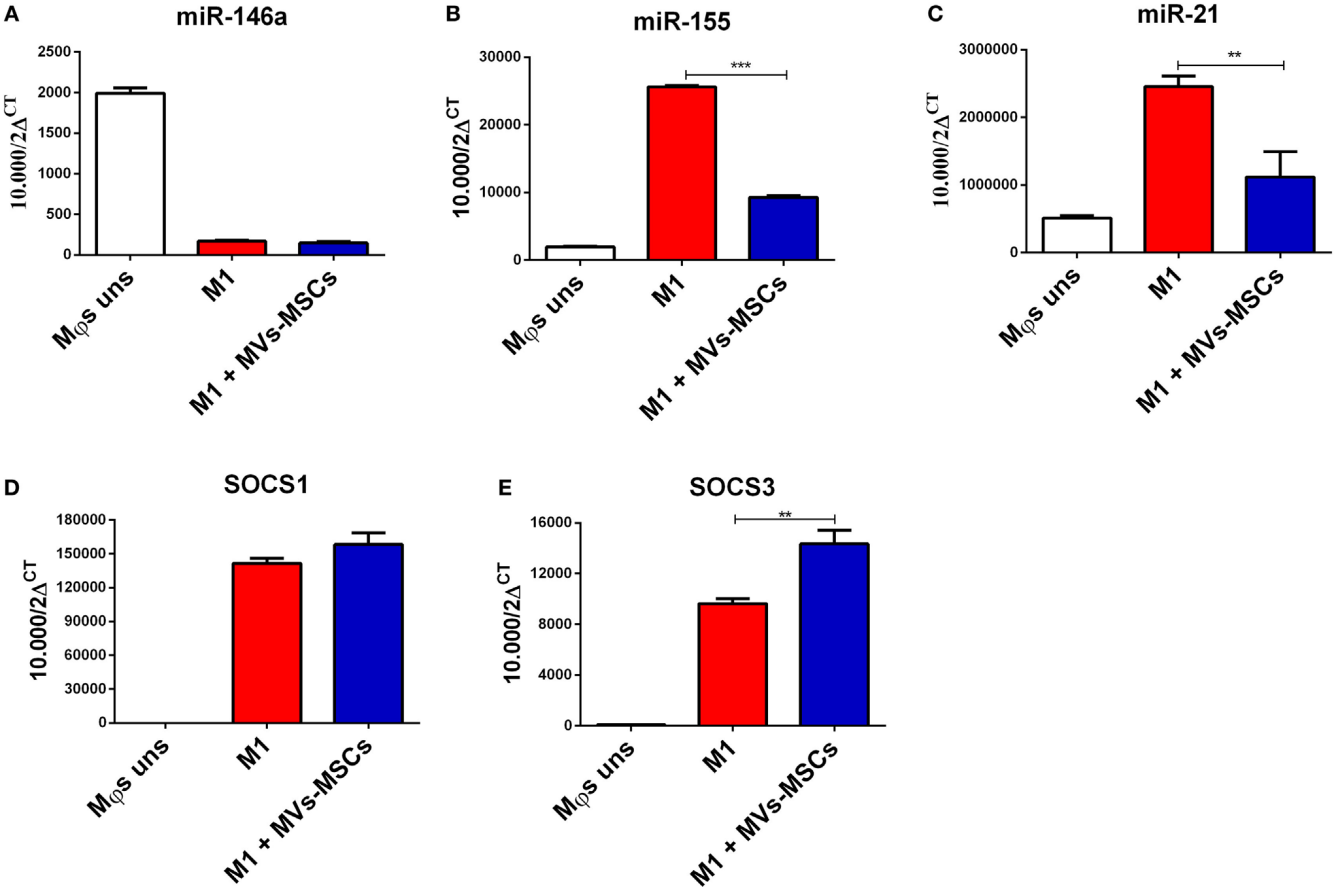
General pro-inflammatory miRNA profile and expression of its predicted target in M1-Mϕs treated or not with MVs-MSCs. **(A)** miR146a expression; **(B)** miR-155 expression; **(C)** miR-21 expression; **(D)** SOCS1 transcriptional level; and **(E)** SOCS3 transcriptional level. The MVs-MSCs treatment downregulates the inflammatory miRNAs expression, as well as of its predicted targets genes in M1-Mϕs (*P < 0.05, **P < 0.01, ***P < 0.001).

After we had identified that MVs-MSCs exerted a regulatory effect on M1-Mϕs through modulation of mRNAs and cell signaling proteins, we studied the ability of MVs-MSCs to modulate some important miRNAs related to M1/M2 phenotype (Figure 4). Analysis of these miRNAs showed that miR-155 and miR-21 were significantly more expressed in M1-Mϕs than in unstimulated Mϕs, considering that miR-146 expression, a regulatory miRNA, was not different between both Mϕs groups (Figures 4A–C). Surprisingly, we detected that MVs-MSCs-treated M1-Mϕs reduced miR-21 and miR-155 expression when compared with M1-Mϕs alone (Figures 4B,C). Thus, these data show that MVs-MSCs may modulate M1-Mϕs *via* downregulation of miR-155 and miR-21 but not by upregulation of miR146a.

In the next step, we set to investigate the predicted target genes of MVs-MSCs-modulated miRNAs in M1-Mϕs. In this sense, we evaluated the expression of SOCS1 and SOCS3 molecules, which possess immunomodulation potential and have been reported as potential targets of these altered miRNAs (Figures 4D,E) (27, 28). Indeed, M1-Mϕs exposed to MVs-MSCs increased the expression of SOCS3 transcript, suggesting that MVs-MSCs upregulate SOCS3 expression by downregulating its associated miR-155, which is also related to M1 activation (Figure 4E). In contrast, the expression of SOCS1 was slightly increased in M1-Mϕs when pulsed with MVs-MSCs, but this was not statistically significant (Figure 4A).

### MVs-MSCs Inhibit Macrophage Recruitment and Regulate Pro-inflammatory Microenvironment in Experimental Peritonitis

In order to elucidate the immunoregulatory effect of MVs-MSCs on activated Mϕs *in vivo*, we examined the influence of MVs-MSCs treatment in an experimental model of thioglycollate-induced peritonitis. This classic acute model is characterized by intense Mϕs recruitment to the peritoneal cavity and release of several inflammatory mediators such as IFN-γ, IL-6, MCP-1, IL-17A, and IL-1β (29). We verified 96 h after thioglycollate injection that there was a massive recruitment of CD11b^+^ cells (macrophages) in the peritoneal space (Figure S4A in Supplementary Material). Complementary, we observed a considerable increase in the total number of inflammatory cells in the peritoneal cavity of animals exposed to thioglycollate over the timecourse (Figure S4B in Supplementary Material).

To validate the predominant presence of Mϕs at day 4, we performed H.E. and IHC staining for CD11b, which confirmed the consistent presence of macrophages in the peritoneal cavity (Figure S4C in Supplementary Material). We also observed that around 55% of neutrophils (GR1^+^ cells) were recruited within 24 h of thioglycollate infusion, but these numbers abruptly decreased over time (20% at 48 h and 5% at 72 h) (Figure S4A in Supplementary Material). In contrast, we detected that the number of peritoneal Mϕs increased considerably after thioglycollate infusion, whose highest concentration in the peritoneal cavity (>80% of CD11b^+^ cells) was observed at 96 h. Together, these results show that at day 4 after peritonitis induction there was a predominant population of Mϕs in the peritoneal cavity with little interference of other inflammatory cells. For this reason, this time point was considered to be suitable to investigate the physiological effects of MVs-MSCs on activated peritoneal Mϕs.

**Figure 5:**
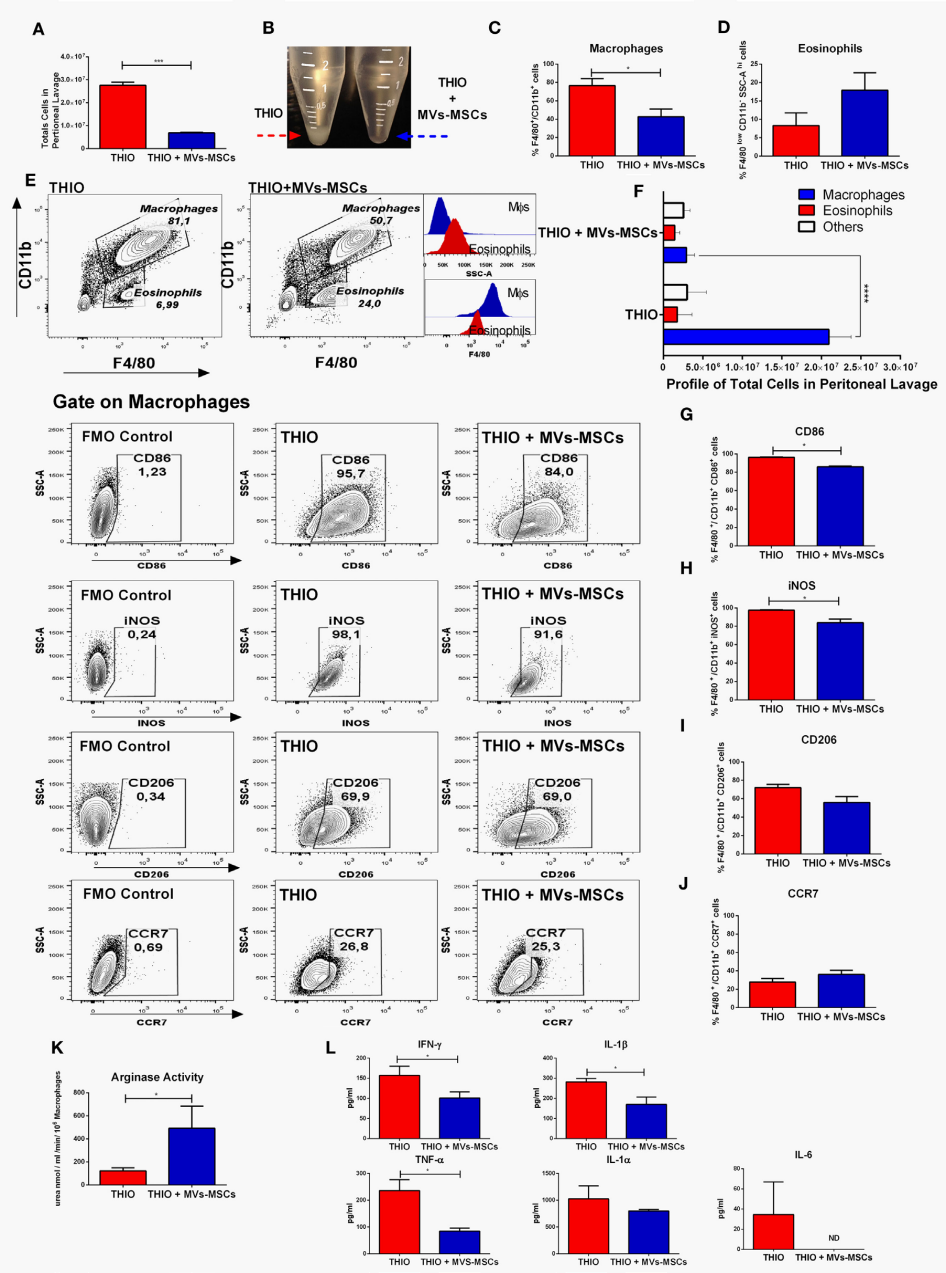
*In vivo* immunoregulatory effect of MVs-MSCs in M1-Mϕs using an experimental model of acute peritonitis. **(A,B)** Total number of peritoneal cells; **(C)** number of peritoneal Mϕs (F4/80^+^CD11b^+^); **(D)** eosinophils frequency; **(E)** representative FACS dot plots showing Mϕs (F4/80^+^CD11b^+^) and eosinophils (F4/80^low^CD11b^-^SSC-A^high^) population between groups THIO and THIO + MVs; **(F)** profile of relative total numbers of Mϕs in peritoneal cavity; **(G)** CD86 expression; **(H)** iNOS index; **(I)** CD206 expression; **(J)** CCR7 expression; **(K)** arginase activity; and **(L)** general profile of Pro-inflammatory cytokines. MVs-MSCs decreased the Mϕs infiltration, increased eosinophils frequency, and promoted an intense immunoregulation of activated peritoneal Mϕs (*P < 0.05, **P < 0.01, ***P < 0.001).

Subsequently, we assessed the role of MVs-MSCs on peritoneal Mϕs in two experimental groups: the control group, which was submitted to thioglycollate and treated with vehicle (named: THIO), and the treated group, which was submitted to thioglycollate and exposed to serial doses of MVs-MSCs (named: THIO+MVs-MSCs). Initially, we compared the total number of peritoneal cells present in both groups. Mice administered MVs-MSCs surprisingly showed a reduction in the absolute number of total cells present in the peritoneal exudate (Figures 5A,B). We observed that MVs-MSCs inhibited the recruitment of approximately 20 × 10^6^ inflammatory cells in peritoneal space (Figures 5A,B). Consistently, we found that mice treated with MVs-MSCs also had reduction in the relative number of peritoneal Mϕs (F4/80^+^CD11b^+^) when compared to the control animals (Figures 5C,E). Next, we built a global profile of the inflammatory cells in the peritoneum, considering the absolute and relative number of cells, and we found that mice treated with MVs-MSCs had 7-fold less peritoneal Mϕs than untreated mice (Figure 5F). These data show that MVs-MSCs were effective o inhibit macrophage recruitment after thioglycollate stimulus (Figure 5F). Information on the absolute and relative numbers of peritoneal Mϕs, as well as of eosinophils in mice not treated with thioglycollate (NON-THIO) can be consulted in the Figures S5A–C in Supplementary Material.

To demonstrated the ability of MVs-MSCs to modulate the function of infiltrating Mϕs in the peritoneal cavity, we evaluated the expression of M1 and M2 markers on these specific Mϕs sub-populations (F4/80^+^CD11b^+^). We found that MVs-MSCs decreased the expression of CD86 in peritoneal Mϕs (Figure 5G). Similarly, Mϕs exposed to MVs-MSCs also reduced the expression of iNOS (Figure 5H). On the other hand, the CD206 and CCR7 levels had a slight reduction after MVs-MSCs treatment but did not reach statical significance (Figures 5I,J). Importantly, when we analyzed the arginase activity in the peritoneal exudate of mice submitted to MVs-MSCs treatment, we found a significant increase when compared to the control group (Figure 5K). Furthermore, we investigated the release of pro-inflammatory cytokines in the peritoneal lavage. Multiplex analysis revealed that, in fact, mice treated with MVs-MSCs have reduced concentrations of most inflammatory mediators in the peritoneal lavage, such as IFN-γ, IL-1β, and TNF-α, while that of IL-1α did not change (Figure 5L). Interestingly, IL-6 was not detected in the washout of the peritoneal cavity from mice treated with MVs-MSCs (Figure 5L). These results suggest that MVs-MSCs were highly effective in regulating classically functional M1-Mϕs during peritonitis *in vivo*, promoting an anti-inflammatory microenvironment.

**Figure 6:**
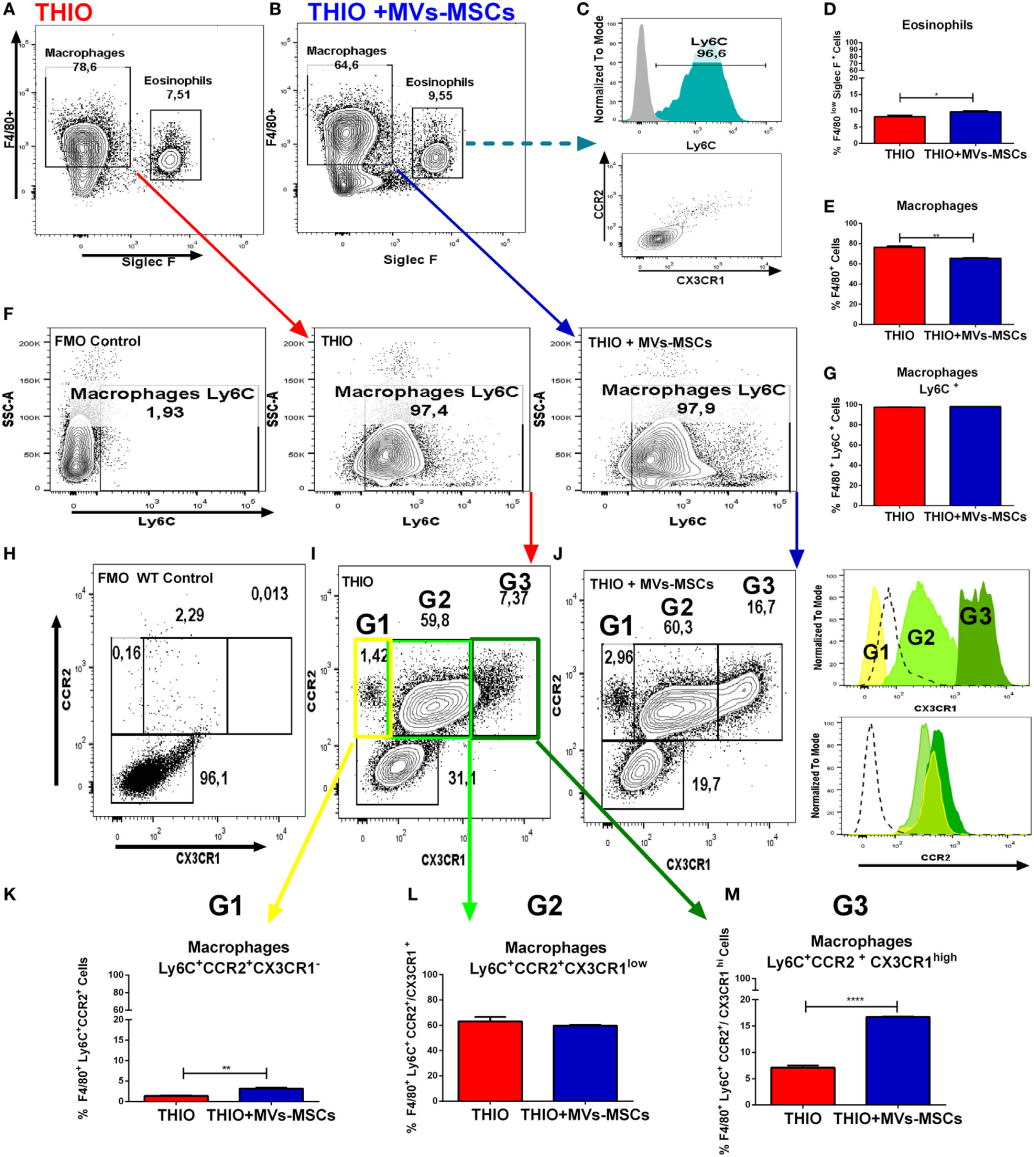
*In vivo* investigation of CX3CR1^+^ macrophage subset population in experimental model of acute peritonitis. **(A,B)** Siglec F^+^ gate strategy to discriminate eosinophil population in peritoneal lavage; **(C)** expression of Ly6C and CCR2/CX3CR1 in eosinophils; **(D)** total eosinophils frequency; **(E)** total macrophage frequency; **(F)** Ly6C^+^ gate strategy to discriminate mature macrophage population in peritoneal lavage; **(G)** total Ly6C^+^ cell frequency; and **(H–M)** identification and expression of CX3CR1 in three specific Mϕs subsets (G1, G2 and G3) in mice treated or not with MVs-MSCs. MVs-MSCs increased the frequency of CX3CR1 in the Ly6C^+^CCR2^+^CX3CR1^high^ Mϕs subset (*P < 0.05, **P < 0.01, ***P < 0.001).

In addition, we also identified the participation of an eosinophil population that has been reported as interfering cells in this model (30). Here, these cells were detected in the peritoneal exudate as a F4/80^low^CD11b^-^SSC-A^high^ gate population in Figures 5D,E, and as F4/80^low^SSC-A^high^SIGLEC F^+^ gate population in Figures 6A,B,D. Although we have observed that MVs-MSCs provided slight increase on relative number of eosinophils in the peritoneum, this difference was not statistically significant (Figure 5D) and, more importantly, these eosinophils were positive for Ly6C but did not exhibit chemokine-associated receptors (CX3CR1 and CCR2) as monocytes, suggesting a non-pivotal participation in inflammatory peritonitis (Figure 6C).

### MVs-MSCs Induce Higher Expression of CX3CR1 in Ly6C^+^CCR2^+^ Macrophages Subsets

Currently, it is known that two different monocyte subtypes have opposite roles in murine inflammation: Ly6C^+^ CCR2^+^ CX3CR1^low^ and LY6C^−^ CCR2^−^ CX3CR1^hi^ monocytes (31). Previous findings suggest that thioglycollate-induced peritonitis promote infiltration of inflammatory Ly6C^+^ monocytes, which subsequently differentiate into peritoneal activated Mϕs (32). In this context, we decided to investigate the influence of MVs-MSCs in the expression of CX3CR1 and CCR2 receptors on activated peritoneal Mϕs. Thus, we performed the thioglycollate-induced peritonitis in heterozygous mice with a florescent gene report for chemokine receptors CX3CR1^+/GFP^ (green) and CCR2^+/RFP^ (red) in inbred mice strains CCR2^+/RFP^ CX3CR1^+/GFP^. Flow cytometry analysis showed that MVs-MSCs were again effective in reducing the relative frequency of peritoneal Mϕs in these fluorescent report mice (Figures 6A,B,E). Next, we found that MVs-MSCs decreased the absolute number of infiltrated cells in the peritoneum cavity (Figure S5F in Supplementary Material), whereas the overall profile of inflammatory cells was similar to the previous experiments depicted in Figure 6F. In addition, we similarly documented some baseline information on macrophage and eosinophil frequencies in NON-THIO mice CX3CR1^+^/^GFP^CCR2^+^/^RFP^ (Figures S5D–I in Supplementary Material).

We have also observed that peritoneal Mϕs (F4/80^+^ gate), both treated and untreated with MVs-MSCs, had high expression of the monocyte marker Ly6C^+^ (Figure 6F,G). These data indicate that MVs-MSCs did not alter the subset of monocytes that infiltrated the peritoneum (Ly6C^+^ or Ly6C^−^) during inflammatory stimulus. Furthermore, we observed the expression of chemokine receptors CCR2 and CX3CR1 in this Mϕs population gated to F4/80^+^Ly6C^+^ (Figure 6H). We detected that Ly6C^+^ Mϕs were positive for CCR2 independently of treatment (Figure 6H, Histograms). This information is consistent with the innate ability of Ly6C^+^ monocytes to exhibit CCR2 in order to migrate from bone marrow to inflammatory tissues. In this context, the MVs-MSCs treatment did not alter the expression of CCR2 in Ly6C^+^ Mϕs (Figure 6H). In addition, peritoneal Mϕs from NON-THIO mice showed low expression of CCR2 and CX3CR1 (Figures S5G,H in Supplementary Material).

Surprisingly, when we evaluated the expression of fractalkine receptor (CX3CR1) on Ly6C^+^ Mϕs population, we could identify three major additional sub-populations based on the fluorescence intensity expression of this molecule: (1) population G1 (Ly6C^+^CCR2^+^CX3CR1^−^ Mϕs), population G2 (Ly6C^+^CCR2^+^CX3CR1^low^ Mϕs), and population G3 (Ly6C^+^CCR2^+^CX3CR1^high^ Mϕs) (Figures 6H–M). Importantly, the frequency of the G3 population was significantly increased in mice treated with MVs-MSCs (Figure 6M), compared to the G1 population, that was slightly elevated, and the G2 population, that did not change. In this context, we may partially conclude that MVs-MSCs regulate peritoneal Mϕs to both M1 and M2 phenotype, as well as upregulate fractalkine receptor (CX3CR1) in specific Mϕs subsets.

## Discussion

Currently, the cell-free therapies with focus on regeneration have been considered to be very attractive and promising approaches (33). Clinically, the use of cell-derived inert particles such as MVs-MSCs to modify target cells by transferring bioactive molecules has strong advantages to traditional interventions, considering they are easily stored, handled, and are safer than parental MSCs (34). With this perspective, in 2011 an intriguing pilot study was conducted with steroid-refractory graft-versus-host diseases patients. Interestingly, that study reported that intravenous administration of MVs-MSCs promoted an improvement in clinical parameters such as diarrhea episodes, skin rash, and mucosal lesions without additional intercurrences (35). Later, the authors showed, using an *in vitro* approach, that the reduction in TNF-α and IFN-γ indexes in NK cells could be partially considered as modulatory mechanism in these MVs-MSCs-treated patients. Considering this evidence, it is plausible that MVs-MSCs could also modulate other immune cells involved in autoimmune processes, such as Th1- and Th17-responses (14, 19). In this sense, we have extensively investigated and demonstrated herein that MVs-MSCs possess *in vitro* and *in vivo* specific abilities to immunomodulate activated Mϕs promoting a pro-resolution microenvironment.

In order to achieve our objective, we first characterized and confirmed the ability of MSCs to regulate M1-Mϕs. This strategy was employed as a quality control step to verify prior of MVs-MSCs isolation its usability. Thus, we can avoid some misconception that cell-derived particles represent only a marginal reference of the biological function status of their parental cells (36). Indeed, our MSCs treatment modulated two pivotal surface antigens required for migration and amplification of the inflammatory response in M1-Mϕs (i.e., CCR7 and CD86) (37, 38). In addition, we also found that CD206 was more expressed in MSCs-treated M1-Mϕs. This modulation was also described by Kim et al. (9), who reported an upregulation of M2 markers (IL-10 and CD206) and downregulation of M1 molecules (IL-12 and TNF-α) in Mϕs cocultured with MSCs. After we had confirmed the functional immunoregulatory abilities of MSCs to modulate M1-Mϕs, we subsequently isolated and characterized MVs-MSCs from the conditioned medium of these cells.

According to the International Society for extracellular vesicles, recent technologies are still inefficient to allow the complete separation of MVs and EXOs with high accuracy (33). However, it is possible to define and identified these sub-populations based on size, phenotypic and structural characteristics. MVs are double membrane particles with sizes between 100 and 1,000 nm and contain high amounts of phosphatidylserine, ceramide, and sphingomyelin. In contrast, EXOs are smaller than MVs (40–100 nm) and contain tetraspanins (e.g., CD9 and CD63), as well as some heat shock proteins. We determined that the population of EVs present in our MVs-MSCs suspension was compatible with an heterogeneous subpopulation enriched with MVs (Figure 1). Thus, our MVs-MSCs expressed surface antigens associated with parental cells and annexin V, which classically define our MSCs-derived particles as predominantly composed by MVs.

One report has demonstrated that MVs-MSCs have elevated distribution of CD44, a receptor related to the ability to incorporate into cell surfaces (39). In this sense, our MVs-MSCs population also exhibited a higher index of CD44, suggesting a promising potential to incorporate and regulate M1-Mϕs. In fact, our findings show that MVs-MSCs were quickly incorporated into the intracytoplasmic region of M1-Mϕs during cocultured experiments, without nuclear co-localization. These data suggest that the preferred site of MVs-MSCs incorporation in Mϕs, in our experimental conditions, would be the cytoplasm. In line with this, previous studies have shown that the therapeutic effect of MVs-MSCs is mainly mediated through transfer and modification of miRNAs that occur primarily in the cytoplasm compartment (40). On the other hand, MVs-MSCs not only can transfer genetic material, but also may transport growth factors and receptors with bioactive functions into target cells. These data support the idea that the elevated M1-Mϕs cellular granularity observed after incubation with MVs-MSCs could be attributed to the transfer of bioactive biomolecules.

In order to investigate the functional immunoregulatory potential of MVs-MSCs, we reported that MVs-MSCs were effective in downregulating M1 markers (i.e., CCR7, IL-1b IL-6, and NO) and subsequently upregulating M2 molecules (IL-10 and CD206) in M1-Mϕs. Surprisingly, M1-Mϕs when exposed to MVs-MSCs exhibited higher arginase activity, which has been recently shown to be a key enzyme associated with M2 regulatory phenotype. This enzyme catalyzes the conversion of l-arginine to l-ornithine, thus suppressing NO production, a pro-inflammatory mediator (41), what was indeed detected in our coculture experiments with MVs-MSCs-modulated M1-Mϕs. Furthermore, arginase also has additional pleiotropic functions such as being a main mediator of cell proliferation, of tissue healing and of inhibition of T lymphocytes (41, 42).

After verifying that MVs-MSCs promoted a M2-like phenotype in activated M1-Mϕs, we attempted to elucidate the mechanism involved in this process. We set out to study the main miRNAs that regulate the Mϕs polarization. Our results show that MVs-MSCs promoted the reduction of miRNAs associated with M1 profile (40) such as miR-21 and miR-155. Importantly, the main function of miR-155 is to suppress IL-13 receptor expression, which regulates the activation of inflammatory Mϕs (40). Moreover, we found that SOCS3 protein, a specific target of miR-155 and miR-21, had its expression reduced in MVs-MSCs-treated M1-Mϕs. In line with this, we further detected the lack of IL-6 expression, a strong regulator of SOCS3, on M1-Mϕs exposed to MVs-MSCs both *in vivo* and *in vitro* experiments. The possible explanation for this molecular interplay would be the fact that SOCS3 is a strong inhibitor of the STAT6 signaling pathway, thus interfering with IL-6 expression, which seems to be regulated downstream by miR-155 (43). Thus, this information suggests that the reduction of miR-21 and miR-155 could be the mechanism by which M1-Mϕs exhibit M2-like phenotype.

To investigate the immunomodulatory role of MVs-MSCs *in vivo*, we evaluated the impact of MVs-MSCs treatment in an experimental model of acute peritonitis. We detected that MVs-MSCs injection promoted a significant reduction in the global number of infiltrating Mϕs into the peritoneal cavity with downregulation of inflammatory markers (CD86 and iNOS) and upregulation of arginase activity. These results complement our *in vitro* previous investigation, demonstrating the functional ability of MVs-MSCs to also regulate M1 peritoneal Mϕs. Supporting this evidence, we detected that pro-inflammatory cytokines such as IL-1β, IFN-γ, IL-6, and TNF-α were broadly reduced in peritoneal Mϕs from mice treated with MVs-MSCs. Additionally, we observed that MVs-MSCs also expressively increased the expression of CX3CR1 in a subpopulation of Mϕs defined here as Ly6C^+^CCR2^+^. Interestingly, Ly6C^+^CCR2^+^ Mϕs are commonly CX3CR1^low^ or negative and are widely associated with pro-inflammatory functions (31). However, in some special situations, it has been documented that Ly6C^+^CCR2^+^ Mϕs may also express CX3CR1^high^, and, in this case, perform anti-inflammatory functions (44). Hence, we can speculate that MVs-MSCs can make use of an alternative mechanism of modulation, that yet needs to be extensively described, involving the upregulation of CX3CR1 in classically inflammatory Mϕs.

In summary, our results indicate that MVs-MSCs have immunomodulatory effects on M1-Mϕs inducing a switch from M1 to M2-like pattern. Thus, our innovative findings suggest that MVs-MSCs can modulate activated Mϕs to an alternative regulatory-like phenotype, thus relevantly contributing to further investigations in the field of inflammatory diseases and tissue regeneration.

## Ethics Statement

All animals’ protocols and experiments were approved by the Ethics Committee on Animal Experimentation of UNIFESP with accession number CEP 685598.

## Author Contributions

Conceptualization: JA and DA; investigation and resources: JA, TB, MA, MAC, RC, AS, MM, ST, MCC, AC, RS-C, and DA; supervision: ÁP-S, DA, and NC; writing: JA and NC.

## Conflict of Interest Statement

The authors declare that the research was conducted in the absence of any commercial or financial relationships that could be construed as a potential conflict of interest.

## Supplementary Material

Video S1 MVs-MSCs incorporation into M1-Mϕs.

Video S2 NON-MVs PKH26 Control.

Video S3 MVs-MSCs PKH26 Stained.
