# Supplementary material for: Mesenchymal Stromal Cell-Derived Microvesicles Regulate an Internal Pro-Inflammatory Program in Activated Macrophages
Source: Front Immunol. 2017 Jul 31;8:881. doi: 10.3389/fimmu.2017.00881 (PMC5535070; doi:10.3389/fimmu.2017.00881)
Supplement: Supplementary file 4 [file Presentation_1.PDF]

frontiers

Supplementary Material

Mesenchymal Stromal Cell-Derived Microvesicles Regulate an Internal Pro-Inflammatory Program in Activated Macrophages.

AUTHORS

Juan S. Agudelo[1*], Tarcio T. Braga[2], Mariane T. Amano[2], Marcos A. Cenedeze[1], Regiane A. Cavinato[1], Amandda R. P. Santos[1], Marcelo N. Muscará[3], Simone A. Teixeira[3], Mario C. Cruz[1], Angela. Castoldi[2], Rita. Sinigaglia-coimbra[4], Alvaro Pacheco-Silva[1,5], Danilo C. de Almeida[2] and Niels O. Câmara[1,2,6].

AFFILIATIONS:

- [1]Department of Medicine, Division of Nephrology, Federal University of São Paulo, Brazil.
- [2]Department of Immunology, Institute of Biomedical Sciences, University of Sao Paulo, Brazil.
- [3]Department of Pharmacology, Institute of Biomedical Sciences, University of Sao Paulo, Brazil.
- [4] Electron Microscopy Center, Federal University of São Paulo, Brazil.
- [5]IEP, Albert Einstein Hospital, Brazil.
- [6]Laboratory of Renal Pathophysiology, Department of Medicine, School of Medicine, University of Sao Paulo, Brazil.

Correspondent author: Niels Olsen Saraiva Camara, MD, Ph.D.

Address: Department of Nephrology, Federal University of Laboratory of Clinical and Experimental Immunology, Brazil, Rua Botucatu 740, 04023-000, Vila Clementino, São Paulo, Brazil, Phone: +55 (11) 55764841. E-mail: niels@icb.usp.br.

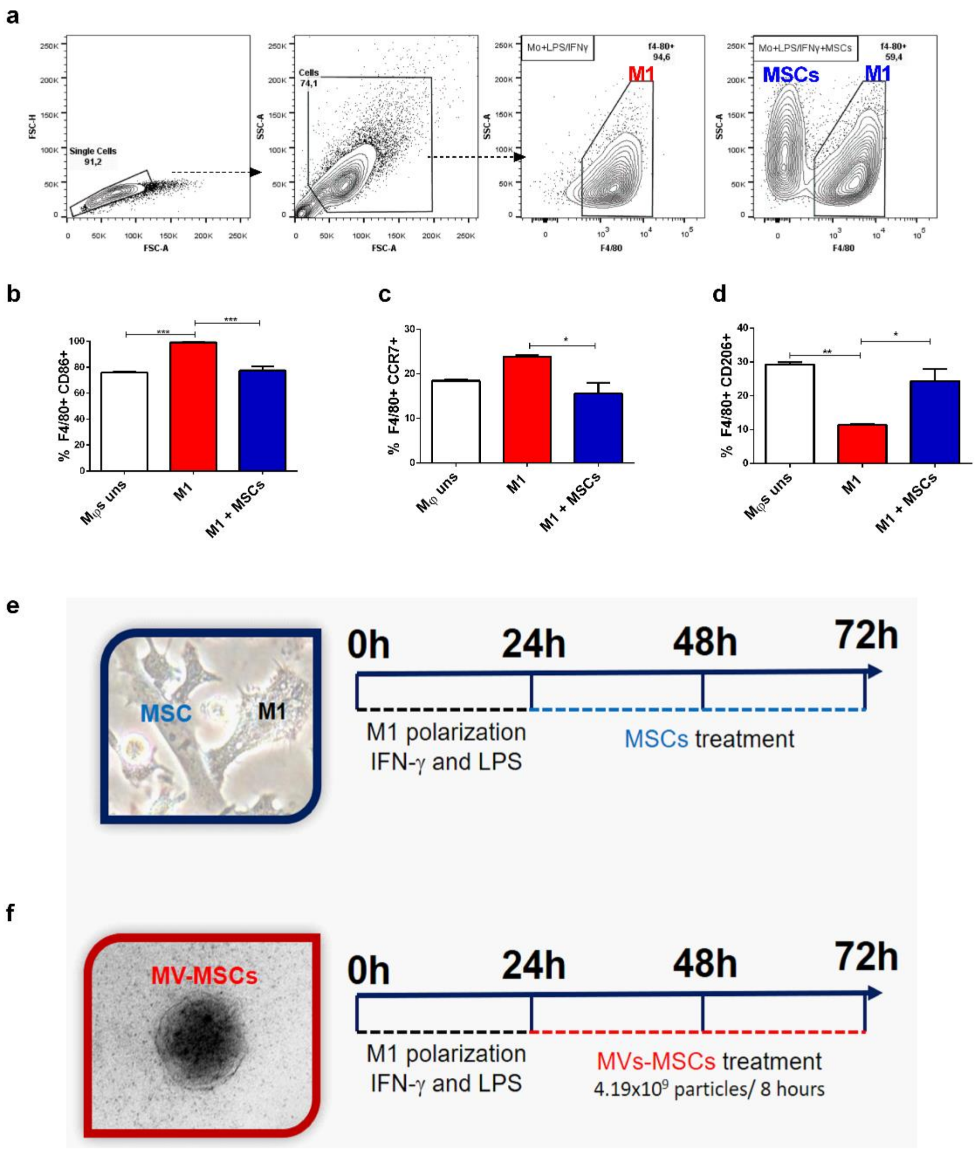

Supplementary Figure 1. Validation of MSC immunomodulation in Mϕs co-cultures and schematic illustration of MSCs or MVs-MSCs treatment in Mϕs cultures. (**a**) F4/80+ gate strategy to discriminate macrophages from MSCs co-cultures; (**b-d**) Expression of M1 (CD86/CCR7) and M2 (CD206) markers in Mϕs in co-culture with MSCs; (**e**) Strategy for MSCs treatment in Mϕs co-cultures and (**f**) Strategy for MVs-MSCs treatment in Mϕs co-cultures (*P<0.05, **P<0.01, ***P<0.001).

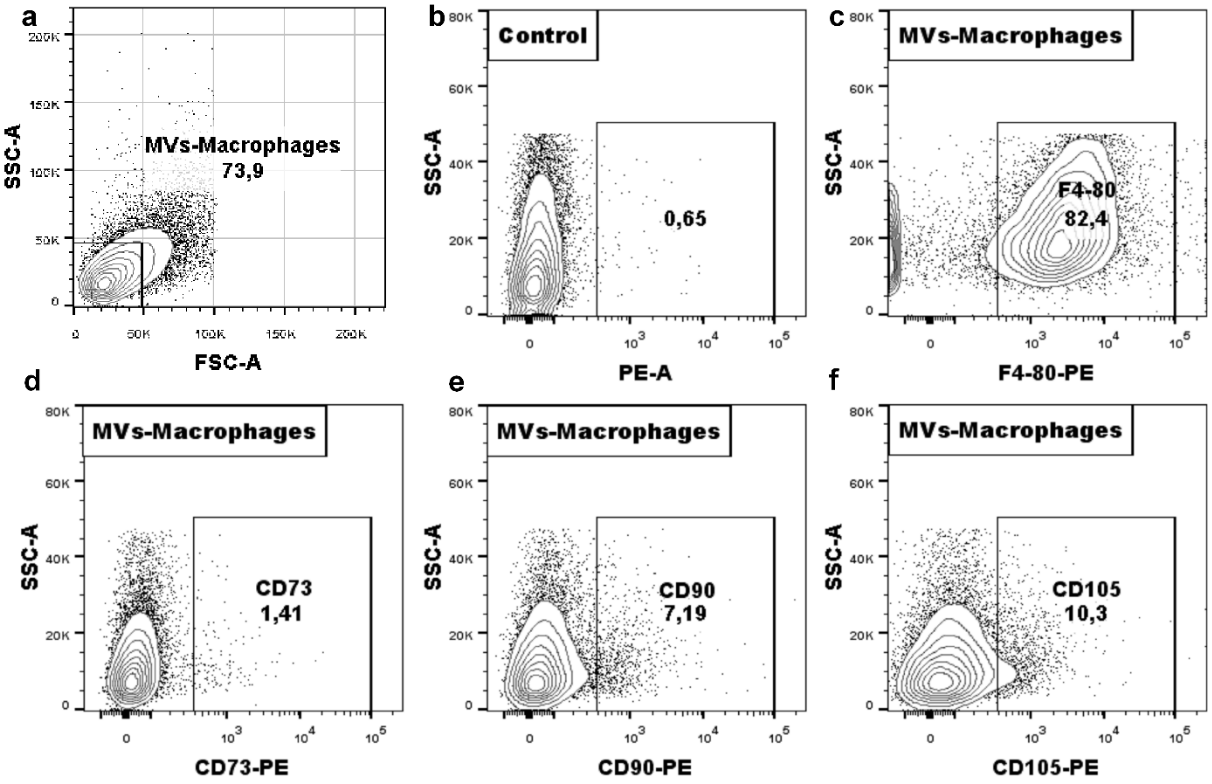

Supplementary Figure 2. Characterization of Mϕs microvesicles (Mϕs-MVs) with MSC surface profile. (a) Mϕs-MVs isolation after ultracentrifugation of Mϕs culture supernatants; (**b**-**c**) F4/80+ gate strategy to discriminate Mϕs-MVs and (**d**) Expression of MSCs surfaces specific markers in Mϕs-MVs. The Mϕs-MVs did not express classical molecules of MSCs suggesting a specific affinity of this molecules for MSCs or MSCs-MVs (*P<0.05, **P<0.01, ***P<0.001).

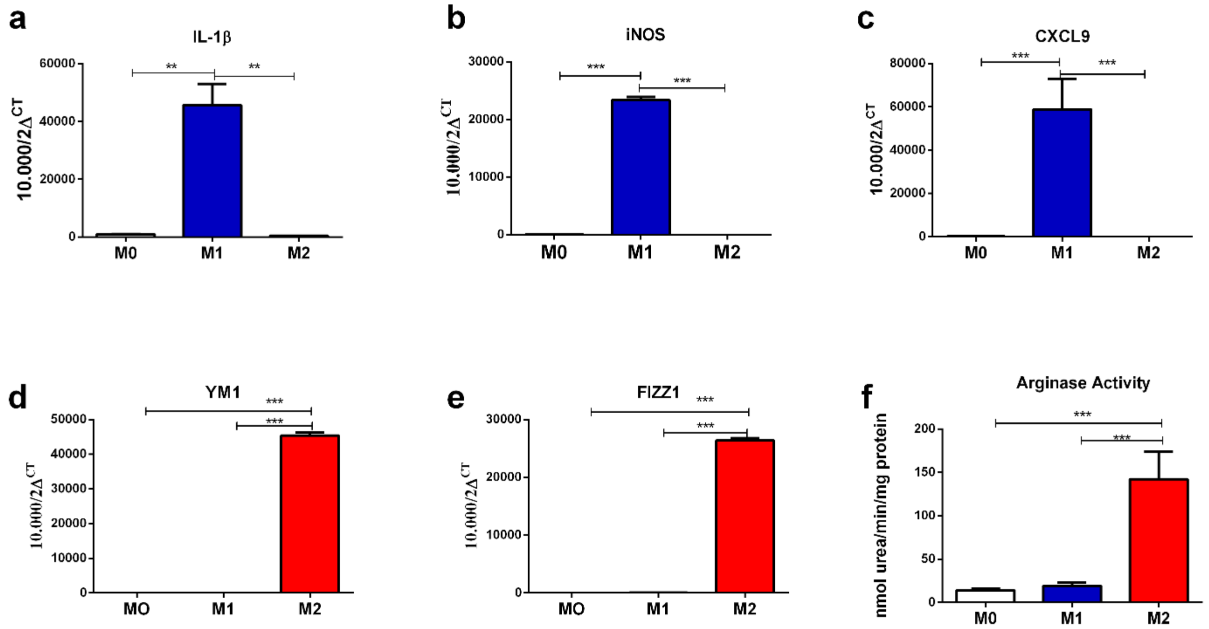

Supplementary Figure 3. M1 and M2 *in vitro* characterization. (**a**) IL-1b expression; (**b**) iNOS expression; (**c**) CXCL9 expression; (**d**) YM1 expression; (**e**) FIZZ1 expression and (**f**) Arginase Activity. It was observed that immature Mϕs can efficiently polarize to M1 and M2 phenotype according with specific stimulus: i) LPS/IFN-γ and ii) IL-4/IL-13, respectively. (*P<0.05, **P<0.01, ***P<0.001).

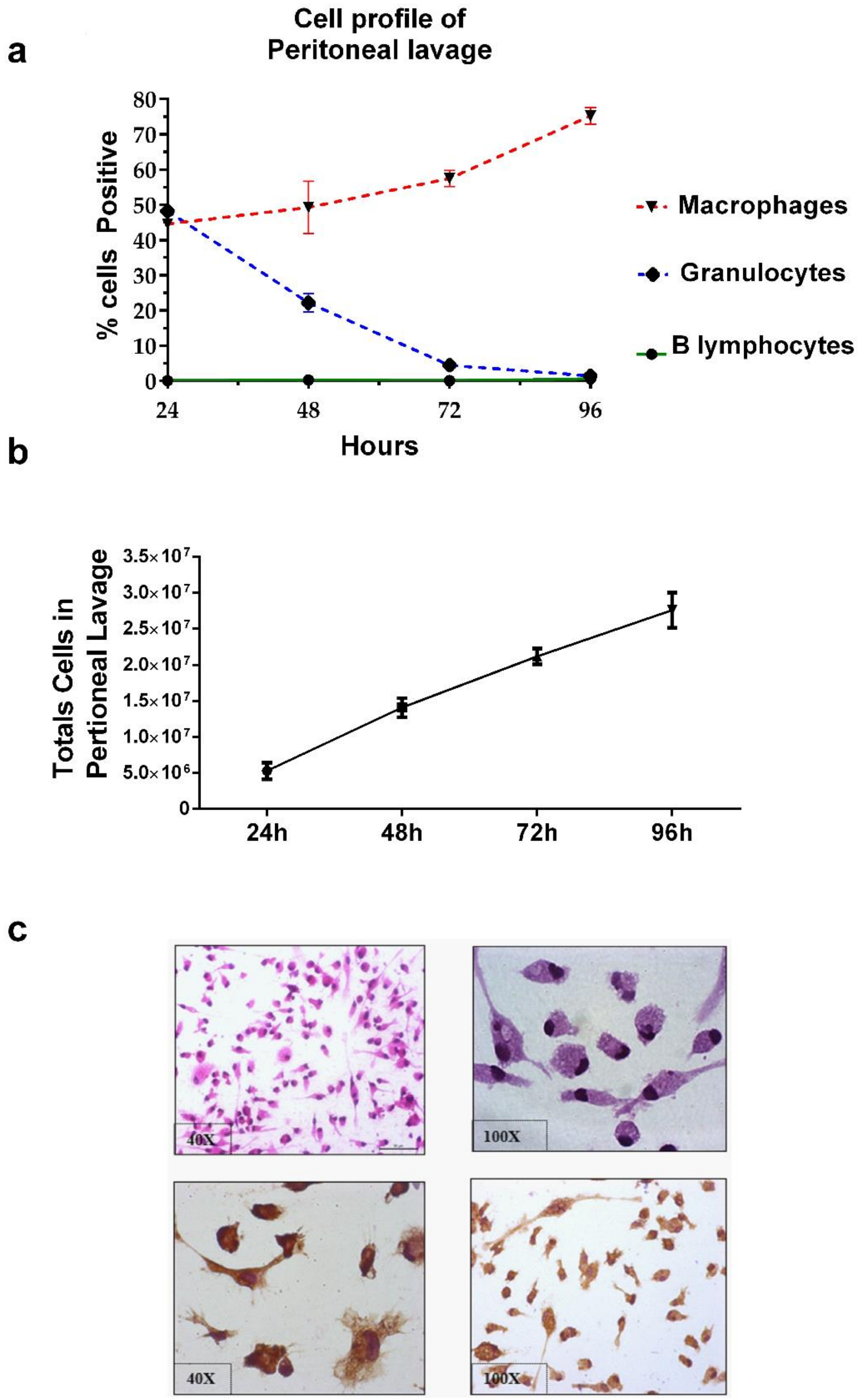

Supplementary Figure 4. Characterization of Mϕs in model of sterile acute peritonitis. (**a**) Cell frequency of immune cells (i.e. granulocytes, macrophages, B Lymphocytes) recruited during acute peritonitis induced by thioglycolate; (**b**) Total number of cells in peritoneal lavage and (**c**) Hematoxylin and Eosin (H&E) staining (purple cells) and CD11b immunohistochemistry (brown cells) of Mϕs at 96 hours post thioglycolate administration (*P<0.05, **P<0.01, ***P<0.001).

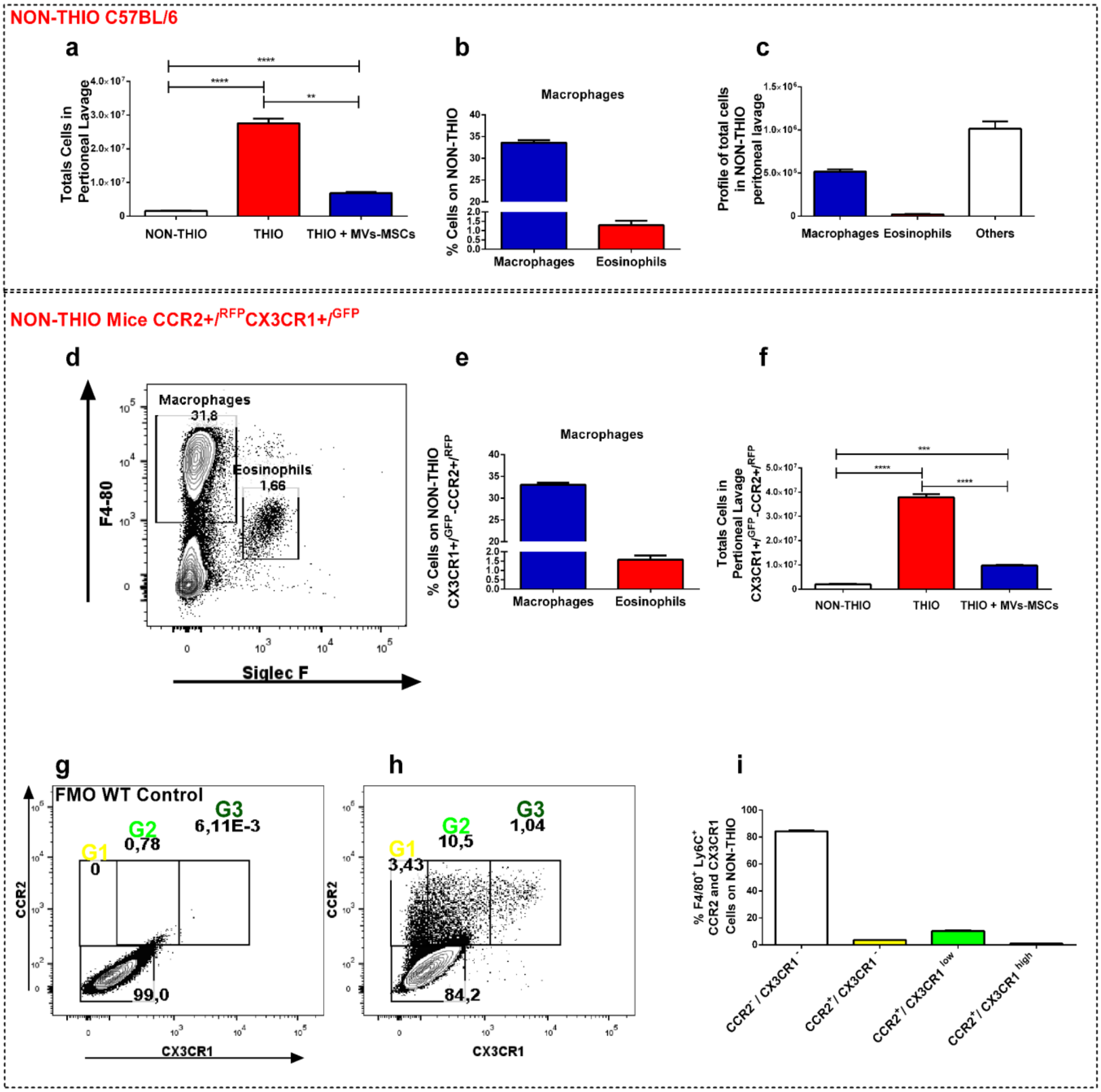

Supplementary Figure 5. General background cell population profile in mice strains C57BL/6 (wild type) and CCR2+/^{RFP}CX3CR1+/^{GFP}. (**a-c**) Frequencies and total number of cells and subsets in C57BL/6 mice injected (THIO) or not with thioglycolate (NON-THIO); (**d-f**) Frequencies and total number of cells and subsets in CCR2+/^{RFP}CX3CR1+/^{GFP} mice injected (THIO) or not with thioglycolate (NON-THIO). (**g**) FMO wt control dot plot and (**h-i**) Frequency of CCR2+/CX3CR1+ cells and its subpopulations (G1, G2 and G3) in NON-THIO CCR2+/^{RFP}CX3CR1+/^{GFP} mice (*P<0.05, **P<0.01, ***P<0.001).
